# Multimorbidity of chronic diseases among adult patients presenting to an inner-city clinic in Ghana

**DOI:** 10.1186/1744-8603-9-61

**Published:** 2013-11-26

**Authors:** Belinda Afriyie Nimako, Frank Baiden, Samuel Oko Sackey, Fred Binka

**Affiliations:** 1 Volta Regional Hospital, Ghana Health Service, Ho, Ghana; 2 Kintampo Health Research Centre, Ghana Health Service, Kintampo, Ghana; 3 University of Ghana School of Public Health, Accra, Ghana; 4 University of Health and Allied Sciences, Ho, Ghana

**Keywords:** Comorbidity, Chronic disease, Non-communicable conditions, Ghana, Africa

## Abstract

**Background:**

Very little is known about multimorbidity and chronic diseases in low and middle income countries, particularly Sub-Saharan Africa, and more information is needed to guide the process of adapting the health systems in these countries to respond adequately to the increasing burden of chronic diseases. We conducted a hospital-based survey in an urban setting in Ghana to determine the prevalence of multimorbidity and its associated risk factors among adult patients presenting to an inner city clinic.

**Methods:**

Between May and June 2012, we interviewed adult patients (aged 18 years and above) attending a routine outpatient clinic at an inner-city hospital in Accra using a structured questionnaire. We supplemented the information obtained from the interviews with information obtained from respondents’ health records. We used logistic regression analyses to explore the risk factors for multimorbidity.

**Results:**

We interviewed 1,527 patients and retrieved matching medical records for 1,399 (91.6%). The median age of participants was 52.1 years (37–64 years). While the prevalence of multimorbidity was 38.8%, around half (48.6%) of the patients with multimorbidity were aged between 18–59 years old. The most common combination of conditions was hypertension and diabetes mellitus (36.6%), hypertension and musculoskeletal conditions (19.9%), and hypertension and other cardiovascular conditions (11.4%). Compared with patients aged 18–39 years, those aged 40–49 years (OR 4.68, 95% CI: 2.98–7.34), 50–59 years (OR 12.48, 95% CI: 8.23–18.92) and 60 years or older (OR 15.80, 95% CI: 10.66–23.42) were increasingly likely to present with multimorbidity. While men were less likely to present with multimorbidity, (OR 0.71, 95% CI: 0.45–0.94, p = 0.015), having a family history of any chronic disease was predictive of multimorbidity (OR 1.43, 95% CI: 1.03–1.68, p = 0.027).

**Conclusions:**

Multimorbidity is a significant problem in this population. By identifying the risk factors for multimorbidity, the results of the present study provide further evidence for informing future policies aimed at improving clinical case management, health education and medical training in Ghana.

## Background

Multimorbidity is defined as the coexistence of two or more chronic diseases in the same individual, and is associated with poorer outcomes and increased healthcare costs [1,2]. Although the health transition is well established in many developing countries, and chronic diseases are already a major health issue, very little is known about the prevalence, forms and clinical outcomes of multimorbidity in these countries.

Much of the information currently available on multimorbidity has been obtained from work undertaken in developed countries. Due to a lack of standard approach to measure multimorbidity, however, the information available on the prevalence and risk factors for multimorbidity in each study depends on the methods used. One study of approximately 900 patients conducted in family practices in Canada showed that the prevalence of multimorbidity (based on the presence of 2 or more chronic health problems) among those aged 18–44 years, 45–64 years, and 65 years and older was 68%, 95% and 99% respectively among female patients, and 72%, 89% and 98% among male patients [3]. In a review of 41 studies by Marengoni et al., mostly undertaken in developed countries, 12 focused on determining the prevalence of multimorbidity and reported prevalences of multimorbidity in older persons of between 55% and 98% [4]. Considering that there is evidence of a positive association between low socio-economic status and multimorbidity coupled with an earlier age of disease onset [5] even in in developed countries, this work needs to be extended to include developing countries and all population subgroups.

One of the few studies undertaken in a developing country context was from Bangladesh, which estimated a prevalence of multimorbidity of 53.8% among persons aged ≥60 years [6]. Despite the variations in the methods of measuring multimorbidity, however, the common position is that multimorbidity is a major health challenge at the primary care level in developed countries [3,7,8] and may be an emerging health problem in developing countries. Knowledge of risk factors for multimorbidity in a given setting is required to guide the development of appropriate interventions for prevention, disease control and clinical case management.

Several studies have shown that the prevalence of multimorbidity increases with age [9,10]. The current evidence on sex as a risk factor for multimorbidity is less clear, however. Although various studies [6,11,12] have identified female sex as a risk factor for the development of multimorbidity, others have suggested that no such association exists [7,10]. It has also been suggested that the relationship between sex and multimorbidity may be dependent on the set of diseases being considered [13]. Socio-economic factors such as low educational attainment and income have been reported as risk factors for multimorbidity [6,12,13]. Schaefer et al. have also shown the lack of an association between marital status and multimorbidity [13]. Although family history of chronic conditions has not been widely studied within the context of multimorbidity, it has been identified as a risk factor for a number of chronic conditions such as ischaemic heart diseases [14], type 2 diabetes and hypertension [15].

Ghana is facing an increasing burden of non-communicable disease [16]. Routine hospital records at Tema General Hospital (TGH) indicate that chronic conditions are consistently within the top 10 causes of in-patient visits, outpatient visits and death [17]. Despite the fact that very little is known about the prevalence of multimorbidity and its risk factors in Ghana, no systematic inquiry into chronic multimorbidity has yet been undertaken in the country. This is indicative of the fact that policies for controlling non-communicable diseases in Ghana are premised on the single disease approach.

We therefore undertook this survey among adults attending a routine outpatient clinic at an urban, government-owned health facility in Ghana to determine the prevalence and determinants of multimorbidity.

## Methods

### Study setting

We undertook the survey among patients presenting to TGH in the Greater Accra Region of Ghana. Tema is located in the southeastern part of the country and has an estimated population of 371,220. This government-run hospital serves as a referral unit for other health facilities in the metropolis and has a high volume of patients. The adult medical outpatient clinic attends to 57% of the annual outpatient caseload of the hospital. A significant proportion of cases seen by this clinic were diagnosed with non-communicable diseases. According to the hospital’s annual reports from 2009 to 2011, hypertension, diabetes mellitus and strokes were among the 10 most common causes of outpatient attendance, admissions and mortality at the hospital.

### Study population

All survey respondents were patients aged at least 18 years who had presented for routine ambulatory outpatient care at the main outpatient department (OPD) of the hospital between 21^st^ May and 8^th^ June 2012. All adult patients presenting to the clinic over the study period were eligible for participation. They were approached by members of the research team and introduced to the aims and objectives of the study. Participants were then invited to be interviewed after providing informed consent.

### Data collection

The survey was carried out using a structured, pre-coded questionnaire administered by health workers who had received training as research assistants for the study. Respondents were assured of the confidentiality of the information they provided, and interviews were conducted in the language in which the respondent felt comfortable. Data was collected on respondents’ demographic and socio-economic characteristics, in addition to self- reported diagnoses (where applicable) and other information provided by respondents on their medical histories. At the end of the clinic day, questionnaires were matched with the patient records, from which we extracted additional information on each respondent’s medical diagnoses and routine medications over a one year period (1^st^ May 2011 was used as the reference date). Only definitive diagnoses were used for the purposes of analysis. Data extraction was carried out by the same two persons throughout the study period.

### Outcome measures

The primary outcome, multimorbidity, was determined using information from participants’ medical records. Multimorbidity was defined as the co-existence of at least two of the 13 pre-selected chronic conditions within the same patient. These 13 conditions were selected following a review of the monthly morbidity returns at the hospital, which identified them as the most prevalent locally. These were: hypertension, diabetes mellitus, musculoskeletal conditions, asthma, sickle cell disease, occupational injuries, malnutrition, intestinal worms, other cardiovascular conditions, chronic eye diseases, skin diseases and ulcers, gastro intestinal diseases and anaemia. While ‘musculoskeletal conditions’ referred to all types of arthritis and chronic back pain (lumbago), and ‘gastrointestinal conditions’ comprised chronic gastritis, tumours and chronic peptic/gastric ulcers, ‘other cardiovascular conditions’ included three possible diagnoses, namely ischaemic heart disease, congestive cardiac failure and stroke. While we used these groupings to conform to the routine hospital records, the specific diseases included in each group were selected based on the existing data at the hospital.

For conditions such as anaemia or skin conditions that could be considered either chronic or acute, 3 months was defined as the minimum duration for a case to be classified as chronic. All conditions were recorded for analysis according to the diagnosis reported in the medical records except for musculoskeletal conditions, cardiovascular conditions and gastrointestinal conditions, which were composite diagnoses as previously described.

Data was inputted into Microsoft Excel 2010 using double-entry methods. The resulting datasets were cleaned and merged to produce one dataset, which was then imported into STATA version 10.0 for analysis.

Following a descriptive analysis, all factors that were significantly associated with the presence of multimorbidity after univariate analysis (p <0.1) were included in the multivariate model to identify independent predictors of multimorbidity. A given risk factor was considered to be significantly associated with multimorbidity if the p-value of the independent association with multimorbidity was < 0.05.

### Sample size

Sample size was estimated on the assumption that 30% of patients presenting at the main OPD would have at least two co-occurring chronic conditions and that 150 patients would be interviewed per day over 10 days. A sample size of 1,500 gave 98% power to detect an effect size of 30% (using a significance level of 5% and a confidence interval of 95%).

### Ethical considerations

Ethical clearance was granted by the Ghana Health Service Ethical Review Committee on Research Involving Human Subjects (ERCRIHS). Permission was also sought from individual participants and hospital authorities to extract information from participants’ medical records, and an approach was adopted to ensure the confidentiality of the information contained within these records. Written informed consent (or by thumbprint if unable to either read or write) was also obtained from all participants.

## Results

### Study participants

From the 1,527 patients interviewed, we retrieved matching hospital records for 1,399 (91.6%) from which we extracted data for analysis. The medical records of the remaining 128 (8.4%) respondents were either unavailable or illegible. While the majority of respondents were female (69.5%), the median age was 52.1 years (37–64 years).

### Prevalence of multimorbidity

The top three conditions among the 13 pre-selected conditions used for defining multimorbidity were hypertension (52.8%), diabetes mellitus (25.4%) and musculoskeletal conditions (17.9%). While 28.3% of study participants had none of the selected conditions, 32.9% had only one. The prevalence of multimorbidity was 38.8% (95% CI 36.3–41.4).

Table 1 summarises characteristics of the study sample and the prevalence of multimorbidity according to participants’ socio-demographic characteristics. Among respondents with multimorbidity, the most common combinations of conditions were hypertension and diabetes mellitus (36.6%), hypertension and musculoskeletal conditions (19.9%), and hypertension and other cardiovascular conditions (11.4%).

**Table 1:** **Distribution of study participants and prevalence of multimorbidity according to participants’ socio**-**demographic characteristics**

**Characteristics**	**Participants (N = 1399)**		**Frequency of multimorbidity**^**ξ**^	
			**(N = 543)**	
	N	%	n	Prevalence (95% C.I.)
**Age (years)**				
18-29	203	14.5	6	3.0 (0.1–5.3)
30-39	211	15.1	29	13.7 (9.1–18.4)
40-49	220	15.7	69	31.4 (25.2–37.5)
50-59	295	21.1	160	54.2 (48.5–60.0)
60 and above	470	33.6	279	59.4 (54.9–63.8)
**Sex**				
Female	972	69.5	401	41.3 (38.2–44.4)
Male	427	30.5	142	33.3 (28.8–37.7)
**Ethnicity**				
Akan	504	36.0	208	41.3 (37.0–45.6)
Ga/Dangme	350	25.0	149	42.6 (37.4–47.8)
Ewe	364	26.0	137	37.6 (32.6–42.6)
Guan	19	1.4	7	36.8 (13.0–60.7)
Mole-Dagbani	66	4.7	17	25.8 (14.9–36.6)
Other	96	6.9	25	26.0 (17.1–35.0)
**Marital status**				
Never married	246	17.6	28	11.4 (7.4–15.4)
Married/living together	790	56.5	327	41.4 (38.0–44.3)
Divorced/separated/ widowed	363	26.0	188	51.8 (46.6–57.0)
**Education**				
No education	319	22.8	152	47.6 (42.1–53.2)
Primary	160	11.4	65	40.6 (32.9–48.3)
Middle/junior high	509	36.4	193	37.9 (33.7–42.1)
Secondary+	411	29.4	133	32.4 (27.8–36.9)
**Occupation**				
Skilled	502	35.9	196	34.7 (30.5–38.8)
Unskilled	897	64.1	347	41.1 (37.9–44.4)
**Total**	**1399**		**543**	**38.8 (36.3–41.4)**

^ξ^Multimorbidity was defined as the presence of at least two of the 13 preselected conditions.

### Risk factors

In the univariate logistic regression models, age, sex, marital status, educational attainment, occupation, ethnicity and a family history of any chronic condition were shown to be associated with multimorbidity to varying extents (Table 2).

**Table 2:** Univariate analysis of potential risk factors for multimorbidity among study participants

**Risk factors**	**Crude OR (95% C.I.)**	**LR p-value**
**Age (years)**		
18-39	1	
40-49	4.95 (3.16–7.75)	
50-59	12.83 (8.47–19.44)	< 0.001
60 and above	15.82 (10.69–23.41)	
**Sex**		
Female	1	
Male	0.71 (0.56–0.90)	0.005
**Marital status**		
Never married	1	
Married/living together	5.50 (3.62–8.35)	< 0.001
Divorced/separated/widowed	8.36 (5.36–13.04)	
**Education**		
No education	1	
Primary	0.75 (0.51–1.10)	
Middle/junior high	0.67 (0.57–0.89)	0.004
Secondary+	0.52 (0.39–0.71)	
**Occupation**		
Unskilled	1	
Skilled	0.76 (0.60–0.95)	0.017
**Family history of chronic conditions**		
No family history	1	
Family history	1.43 (1.16–1.78)	0.001
**Ethnicity**		
Akan	1	
Ga/Dangme	1.05 (0.80–1.39)	
Ewe	0.86 (0.65–1.13)	
Guan	0.83 (0.32–2.14)	0.008
Mole- Dagbani	0.49 (0.28–0.88)	
Other	0.50 (0.31–0.82)	

After adjusting for the effects of those risk factors which were significantly associated with multimorbidity in the univariate analysis, only age, sex and a family history of a chronic condition were found to be independently associated with the risk of having multimorbidity.

Age was the most significant independent risk factor. Increasing age was associated with a higher risk of multimorbidity. Compared with patients aged 18–39 years, older patients were at progressively greater risk of multimorbidity. Estimated odds ratios were 4.68 for patients aged 40–49 years (95% CI: 2.98–7.34), 12.48 for those aged 50–59 years (95% CI: 8.23–18.92) and 15.80 for those aged 60 years and above (95% CI: 10.66–23.42, p < 0.001).

Men were significantly less likely to have multimorbidity (OR 0.71, 95% CI: 0.45–0.94, p = 0.015). In addition, there was a strong association between self-reported family history of any chronic condition and the risk of having multimorbidity (OR 1.43, 95% CI: 1.03–1.68, p = 0.027). Table 3 summarises the output of the multivariate logistic regression model showing the independent associations between these socio-demographic characteristics and multimorbidity.

**Table 3:** Risk factors for multimorbidity in study participants

**Risk factors**	**Adjusted OR**	**95% CI**	Adjusted LR; p-value*
**Age (years)**			
18–39	1	1	
40–49	4.68	2.98–7.34	
50–59	12.48	8.23–18.92	< 0.001
60 and above	15.80	10.66–23.42	
**Sex**			
Female	1	1	
Male	0.71	0.54–0.94	0.015
**Family history of chronic conditions**			
No family history	1		
Family history	1.43	1.03–1.68	0.027

*Odds ratios (*OR*) were adjusted for age, sex, family history, marital status, education, type of occupation and ethnicity (which were found to be significant predictors of multimorbidity in the univariate analysis) using logistic regression. *CI* = Confidence Interval, *OR* = Odds Ratio.

## Discussion

This study is the first to explore multimorbidity in Ghana, and its results will be of importance to health managers in adapting medical education curricula, clinical case management guidelines and the structure of the health system in general to respond to the health transition currently taking place in Ghana. The study has a number of important limitations, however. Being a cross-sectional study, it is unable to establish temporal associations between the socio-demographic factors studied and the occurrence of chronic conditions. It is worth bearing in mind, however, that factors such as sex and family history are not influenced by temporality as they do not change with time.

The fact that data from a single secondary care clinic in an urban setting was used in the analysis limits the extent to which the results of the present study can be generalised to the population as a whole. Our approach of including all patients seen during clinical sessions within a given time period may have led to oversampling of complex patients with several diseases or frequent attendees [18]. Furthermore, although it gives an indication of the burden on the health system, it gives little indication of the health status of the wider community where the true burden of multimorbidity lies. In spite of the advantages of using medical records, the information obtained may be subject to local diagnosing patterns and practices. Given the varying approaches adopted in different studies to explore multimorbidity in different settings, any comparison of the findings of the present study with those of other studies should be made with caution. Consideration should be given to the setting and the issues investigated.

This study has also shown that over a third of the adults over 18 years of age surveyed (38.8%) who attended the medical clinic of the main OPD at TGH during the study period had multimorbidity. Several studies, particularly in developed countries, have determined the prevalence of multimorbidity in a number of different settings using a variety of methods — making comparison difficult [4,18]. The prevalence of multimorbidity estimated in the present study is similar to the 37.1% prevalence reported in Australia, where the study population was sourced from patients sampled from the waiting room of a general practice [7]. However, this figure is lower than that obtained in other hospital-based studies such as the one undertaken by Fortin et al. in Canada, which reported that 9 out of 10 patients had more than one chronic condition [3]. This is expected given that multimorbidity increases with age, and that populations in developed countries have a higher median age. The age-group specific prevalence for persons 60 years and over was 59.4%. This is also similar to the reported prevalence of 58.6% for a sample of people aged 65–94 years in Germany [19]. It is important to note, however, that any comparison of the prevalence of multimorbidity at different sites is limited by differences in methods employed and the age structure of the populations surveyed. Despite these difficulties in making comparisons, this study has provided evidence that, even in this setting, multimorbidity is an important challenge that both health practitioners and the health system as a whole urgently need to address. It makes a case for consideration of multimorbidity in medical training, and in designing guidelines and strategies for prevention and care.

It would be useful, however, to determine prevalence using community-based study to obtain more accurate estimates of the burden of disease on a population level. This is especially important in the Ghanaian context, where awareness of specific diseases among the general population has been described as low [20,21], resulting in late presentation by affected patients. Age was the most significant socio-demographic risk factor for multimorbidity in this study. Notably, over half of the patients above 60 years had multimorbidity. Our results mirror those of a number of studies [12,22]; in further support of the evidence for positive association between prevalence of multimorbidity and increasing age which has been described as almost conclusive [18]. While this study highlights the importance of giving increased consideration to persons with multimorbidity, particularly the elderly, providing a continuum of care and integration should be considered a priority [23].

Females were at an increased risk of multimorbidity, irrespective of age. Our findings support those of previous studies [6,11,12], but contrast with others [7] which did not find an association between multimorbidity and sex. Reasons for this increased risk in females may be due to genetic factors, living and working environments, life events, behavioural risk factors or the general risks associated with low socio-economic status [6]. Consequently, further work is needed to identify which of these factors are most critical. The benefits of prevention efforts may be most beneficial among females, particularly young adults.

Socio-economic status (SES) is usually measured by determining level of education, income, occupation or a composite of these indicators [24]. In this study, both education and occupation were associated with multimorbidity in the univariate logistic models only, and did not show any association with multimorbidity after adjusting for other factors. Similar conclusions were drawn by a Canadian study [25], which found only a crude association between education and multimorbidity. The absence of a clear association could be due to the methods of assessment used, or due to the interplay between the influences of a sedentary lifestyle with higher SES, greater health awareness and improved access to health care — or the reverse scenario for participants with low SES. Finally, the likelihood of having multiple chronic conditions increased with a positive family history of any chronic disease. This may be due to genetic, behavioural or environmental factors common to members of the same family.

This study provides a basis for further studies to inform future policies to reduce the burden of multimorbidity, particularly in an African context.

## Conclusion

The present study shows that multimorbidity exists in around four out of every 10 patients aged 18 years and above presenting to a hospital in urban Ghana, and that the risk factors for its occurrence are age, sex, and a family history of chronic disease. Further research is needed to establish the prevalence of multimorbidity across a wider range of health facilities and in different populations throughout the country. The findings of this study support the current evidence that non-communicable diseases represent a major health issue in Ghana.

## Abbreviations

ERCRIHS: Ghana Health Service Ethical Review Committee on Research Involving Human Subjects; OPD: Outpatient department; SES: Socio-economic status; TGH: Tema General Hospital.

## Competing interests

The authors declare that they have no competing interests.

## Authors’ contributions

BN designed and carried out the study as part of her Master of Public Health Dissertation work under SS’ supervision with substantial contributions to the design of the study from FBi. BN wrote the first draft which was critically reviewed by FBa, who also reviewed subsequent drafts. All authors reviewed the manuscript and have given final approval of the version published.
